# Alterations in the expression of vascular endothelial growth factor in the rat brain following gamma knife surgery

**DOI:** 10.3892/mmr.2014.2520

**Published:** 2014-08-27

**Authors:** LEI CHENG, LIN MA, HECHENG REN, HONGWEI ZHAO, YIQIANG PANG, YONGHENG WANG, MING WEI

**Affiliations:** 1 Department of Neurosurgery, Tianjin Huanhu Hospital, Tianjin 300060, P.R. China; 2 Department of Neurosurgery, Jixian People’s Hospital, Tianjin 301900, P.R. China; 3 Department of Neurosurgery, Fourth Hospital of Baotou, Baotou, Inner Mongolia 014030, P.R. China; 4 Department of Neurosurgery, First Hospital of Qinhuangdao, Qinhuangdao, Hebei 066000, P.R. China; 5 Department of Neurosurgery, Second Hospital of Tianjin Medical University, Tianjin 300211, P.R. China

**Keywords:** brain edema, Gamma knife surgery, vascular endothelial growth factor

## Abstract

Gamma knife surgery (GKS) is used for the treatment of various brain diseases. However, the mechanisms underlying brain injury following irradiation remain to be elucidated. Given that vascular endothelial growth factor (VEGF) is closely associated with pathological angiogenesis and the permeability of the blood brain barrier (BBB), the present study was designed to analyze temporal alterations in VEGF expression in the cerebral cortex and the effect of VEGF on cerebral edema in rats following GKS. Adult male Wistar rats were subjected to GKS at maximum doses of 60 Gy. Animals were sacrificed between 4 and 24 weeks after GKS. Immunohistochemistry, enzyme-linked immunosorbent assay and reverse transcription-polymerase chain reaction (RT-PCR) were employed for detecting VEGF expression. The vessel density was measured by CD31^+^ cell count and vascular structures were examined using electron microscopy. Brain water content and BBB permeability were measured in the present study. VEGF expression in the irradiated cortex progressively increased until 16 weeks after GKS when the maximal expression was reached, and then gradually decreased to the control level 24 weeks after GKS. These findings were confirmed by RT-PCR. A mild decrease in vessel density was observed 4 weeks after GKS, followed by an increase in vessel density between 8 and 20 weeks later. Furthermore, previous studies also demonstrated vascular damage, opening of the BBB and an increase in brain water content occurring simultaneously. To the best of our knowledge, these data demonstrated for the first time dynamic changes in VEGF expression following GKS and also suggest the importance of VEGF expression in pathological angiogenesis and edema formation following GKS.

## Introduction

Gamma knife surgery (GKS) is the stereotactic delivery of a single high-radiation dose as an alternative to neurosurgical open surgery. The frequency of GKS use is expanding in the treatment of vascular malformations, brain tumors and functional brain diseases (1–3). During the course of application of a therapeutic dose of radiation to the lesion, a certain volume of surrounding normal tissue, although small, receives a destructive radiation dose. This leads to the occurrence of a variety of clinical complications, among which the delayed formation of edema in the radiosurgery bed is the most common complication associated with normal tissue radiation injury (4). However, the mechanisms producing brain edema following Gamma knife irradiation remain to be elucidated.

Brain edema formation is closely associated with pathological angiogenesis. Vascular abnormalities are also most often observed in central nervous system (CNS) damage resulting from radiosurgery, which includes endothelial cell swelling, vessel dilation and basement membrane thickening, as well as changes in vascular permeability (5,6). Increased vascular permeability resulting from radiation-induced pathological angiogenesis is one of the main causes of radiation-induced edema (7). Furthermore, damage to the microvasculature in the CNS is the primary event that is causative in the subsequent development of late effects, thus the present study aimed to examine the expression of growth factors associated with angiogenesis.

Vascular endothelial growth factor (VEGF) is a member of a family of angiogenesis-associated growth factors (8). It is a highly conserved 36–46 kD diametric glycoprotein with potent and specific activity for endothelial cell proliferation and vascular permeability to water and large-molecular-weight proteins. In pathological states, VEGF is upregulated in response to increased metabolic demand (9). The biochemical properties of VEGF provide potential therapeutic anti-VEGF agents to control edema (10). There have also been several studies investigating how VEGF contributes to pathological angiogenesis and edema formation in brain tumors, stroke and traumatic brain injury, however, information on GKS is extremely limited (11). Previous studies have revealed that radiation-induced increases in VEGF expression in white matter following conventional radiotherapy were present in regions of blood-spinal cord barrier disruption and tissue hypoxia (12). The radiobiological changes associated with radiosurgery are different to the changes observed following conventional radiotherapy with a lower dose. Dynamic alterations in the expression of VEGF following GKS *in vivo*, however, have yet to be determined.

In the current study, in order to identify the effects of VEGF on pathological angiogenesis and brain edema following GKS, whether VEGF expression is upregulated in normal tissue by gamma knife irradiation was investigated and subsequently fluctuations in the expression of VEGF during the course of gamma knife irradiation was monitored.

## Materials and methods

### Animal protocol

A total of 96 male Wistar rats weighing between 200 and 240 g were housed in cages (two rats per cage) and maintained in environmentally controlled rooms (22–24°C) with a 12-h light/dark cycle. Experiments involving animals were approved by the Animal Care and Ethics Committee of Tianjin Medical University (Tianjin, China). A maximum dose of 60 Gy was administered into the right parietal cortex via the Leksell gamma knife model C (Elekta Instrument AB, Stockholm, Sweden) by using a 4-mm collimator (Elekta Instrument AB, Stockholm, Sweden) as described previously (13). The selection of the radiation dose was based on previous studies (14,15). A rat anesthetized with 10% chloraldurat (3 ml/kg) was fixed in a stereotactic brain frame. Following obtaining high-resolution magnetic resonance (MR) images, the center of the irradiation area was calculated with reference to a standard rat stereotactic atlas (16) and the cerebral structures visible on the MR images. Leksell Gamma Plan software (Elekta Instrument AB) was used to attain target localization for the radiosurgery. The control animals were treated identically but did not receive any radiation.

### Histology and immunohistochemistry

At 4, 8, 12, 16, 20 and 24 weeks post-irradiation, the rats were perfused transcardially with normal saline followed by 4% buffered paraformaldehyde (PFA) under intraperitoneal anesthesia (n=4 per each time point). The brain was removed and the brains were excised. Following fixation in 4% phosphate-buffered PFA, the brains were cut transversally into 2 mm-thick slices and embedded in paraffin. Sections (4 μm thick) were cut and mounted on positively charged glass slides. The avidin-biotin complex (ABC) method (Vectastain Elite ABC kit; Vector Laboratories, Burlingame, CA, USA) was used for the CD31 and VEGF primary antibodies (Santa Cruz Biotechnology, Inc., Santa Cruz, CA, USA). The sections were boiled at 95–100°C in citrate buffer (pH 6.0) for antigen retrieval. Sections were treated with 1.5% normal horse or goat serum for 30 min, followed by incubation overnight at 4°C with mouse polyclonal primary antibodies (1:100). Following incubation with diluted biotinylated rabbit anti-mouse secondary antibody, sections were incubated with ABC reagent for 30 min, and 0.015% hydrogen peroxide and 0.05% diaminobenzidine for 5–10 min. Nuclear staining was performed with hematoxylin. The CD31^+^ structure was quantified from 10 random ×200 fields in a blinded fashion and was converted to square millimeters.

### Enzyme-linked immunosorbent assay (ELISA)

Under deep anesthesia, animals were sacrificed at designated time points. Following decapitation, the brains were rapidly removed and the ipsilateral parietal cortex receiving GKS was dissected freely. The cortical tissue samples were weighed and homogenized on ice in four volumes of extraction buffer consisting of 150 mmol/l sodium chloride, 50 mmol/l mm tris aminomethane hydrochloride and 1% Triton X-100 (pH 7.2) with a proteinase inhibitor cocktail (Sigma-Aldrich, St. Louis, MO, USA). These were then agitated for 90 min at 4°C and centrifuged for 15 min at 3,000 × g. The resulting supernatants were aspirated and VEGF concentrations were determined by ELISA, according to the manufacturer’s instructions (R&D Systems, Minneapolis, MN, USA). Cytokine concentrations were standardized against total protein concentration in the homogenates using a BCA protein assay BCA (tm) Protein Assay kit (Pierce, Rockford, USA).

### Reverse transcription-quantitative polymerase chain reaction (RT-qPCR)

For analysis of mRNA levels, brains between 4 and 24 weeks post-irradiation were removed and the irradiated ipsilateral cortex was carefully dissected and snap-frozen in liquid nitrogen. Total RNA was isolated according to the manufacturer’s instructions of the TRIzol^®^ reagent (Invitrogen Life Technologies, Carlsbad, CA, USA) including DNase treatment. For qPCR, reverse transcription of RNA samples was performed using a PrimeScript™ RT reagent (Takara Bio Inc., Shiga, Japan). The relative quantity of target mRNA was determined using SYBR^®^ Premix Ex Taq™ II (Takara Bio Inc.) according to the manufacturer’s instructions. The following PCR primers were used: VEGF, forward 5′-GCTCTCTTGGGTGCACTGGA-3′ and reverse 5′-CACCGCCTTGGCTTGTCACA-3′. Cycle thresholds (CT) for single reactions were determined using MyiQ software (Bio-Rad, Hercules, CA, USA) and the target genes were normalized against GAPDH. The 2^−ΔΔCT^ method was used to calculate relative changes in gene expression.

### Transmission electron microscopy

The rats were perfused with 4% PFA and 0.125% glutaraldehyde under intraperitoneal anesthesia with 10% chloraldurat (3 ml/kg). The radiation target was identified by the rat stereotactic atlas and using a previously described method (14). The parietal cortical tissue specimens at the site of radiation were immediately excised and 1 mm^3^ tissue blocks were cut out, followed by fixation in a solution of 2% PFA and 2.5% glutaraldehyde in 0.1 mol/l phosphate buffer. The samples were dehydrated in a series of ethanol dilutions and transferred to 100% propylene oxide for 15 min, followed by graded resin infiltration and embedding. Ultrathin sections were prepared on a Leica Ultracut ultramicrotome (Leica, Bensheim, Germany) using a 45 degree diamond histoknife. Images were captured using an Hitachi H7600 transmission electron microscope (Hitachi, Tokyo, Japan).

### Evans Blue (EB) extravasation

A 2% (w/v) solution of EB at 4 ml/kg was injected intravenously and allowed to circulate for 30 min. The rats were sacrificed and the brains were dissected. Quantification of the brain EB extravasation was performed using a spectrophotometer (Tecan, Männedorf, Switzerland). Brains were homogenized by vortexing in 250 μl phosphate-buffered saline (PBS) for 2 min. Subsequently, 250 μl of 60% trichloroacetic acid was added and the samples were vortexed for 2 min. Following cooling for 30 min, the samples were centrifuged for 5 min at 10,000 g. Absorbance readings were measured at 620 nm and EB extravasation was expressed as ng of EB per milligram of brain tissue.

### Fluorescent microscopy for EB extravasation

EB can be visualized as bright orange under a fluorescent microscope (Leica DMLB; Leica, Wetzlar, Germany). In order to process fluorescence microscopy, the brains were perfused via the ascending aorta with 5 ml saline, followed by 5 ml of 4% PFA in 0.1 m PBS at pH 7.4. The brains were removed and stored overnight in the same solution. The brains were immersed in 30% sucrose in 0.1 m PBS for 48 h and then frozen in Optimal Cutting Temperature medium (Sigma) and stored at −80°C. The EB extravasation was observed in the cryostat sections (20 μm) using a fluorescent microscope (Leica DMLB; Leica, Wetzlar, Germany).

### Water content analysis by dry/wet weight measurements

Following anesthesia, rats were euthanized by cervical dislocation, the brains were collected and the irradiated cortexes were separated and carefully dissected. Tissues were weighed immediately and dried in a vacuum for up to 2 weeks. The weights were collected throughout the drying period until the final dry weight was established. Water content was calculated as follows: Water content = (wet weight − dry weight) / wet weight × 100%.

### Statistical analysis

Data are expressed as the mean ± standard deviation. Comparisons among multiple groups were performed by analysis of variance with Dunnett’s post hoc tests. Comparisons between the two groups were performed by Student’s t-test. P<0.05 was considered to indicate a statistically significant difference.

## Results

### VEGF^+^ cells in irradiated brain tissue

**Figure 1:**
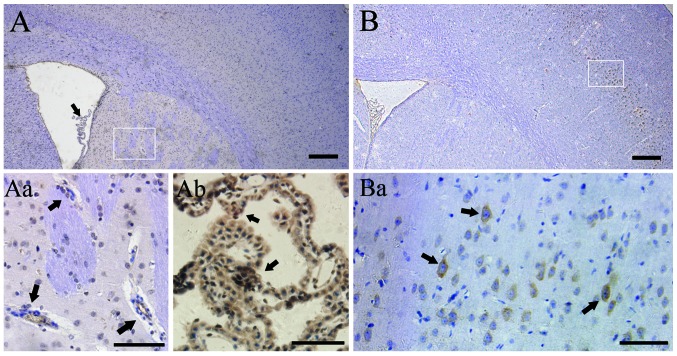
Expression of VEGF by immunochemistry. This analysis demonstrated that few cells were stained by VEGF in the cortex of (A) sham-surgery rats, and VEGF expression was only observed in (Aa) periventricular regions and (Ab) choroid plexuses. However, VEGF was significantly expressed in the cortex of the irradiated hemisphere 8 weeks after GKS (B) and was predominantly located in neurons (Ba). VEGF, vascular endothelial growth factor. Magnification: A, ×40; a1, ×400; a2, ×400; B, ×40; b, ×400,

In the control group, positive staining for VEGF was not observed in the cortex (Fig. 1A), but rather in choroid plexuses (Fig. 1Aa) and endothelium of the periventricular region (Fig. 1Ab). In comparison, the prominent VEGF^+^ cells were detected in the superficial neuronal layers of the injured ipsilateral cortex (Fig. 1B and Ba) 8 weeks after radiation. The results demonstrated a dynamic change in VEGF expression. There were hardly any immunopositive stained cells in the irradiated right parietal cortex 4 weeks after GKS. The number increased rapidly 8 weeks after GKS (25.500.96 cells/mm^2^; P<0.001; Fig. 3E) and then reached a plateau at ~16 weeks after radiation (45.00±2.45 cells/mm^2^; P<0.001; Fig. 1A), prior to decreasing to baseline. In comparison, VEGF^+^ cells remained constantly lower in brain tissue from the control rats, and no significant change was identified in VEGF^+^ cell distribution and quantity in the control group during the same follow-up period (P>0.05).

### Quantitative concentration analyses of VEGF over time following irradiation

**Figure 2:**
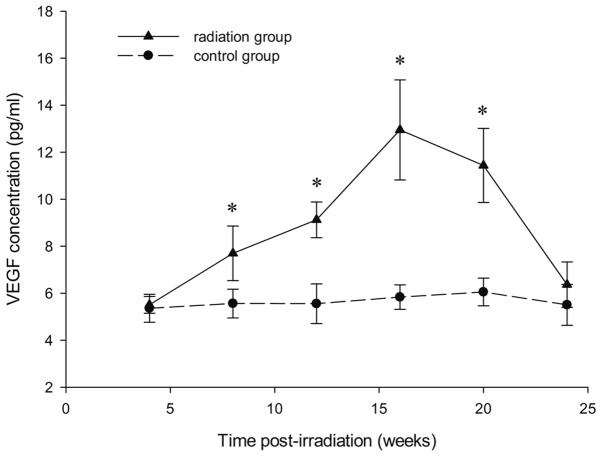
Alterations in the concentration of VEGF up to 24 weeks after GKS. The data are presented as the mean ± standard deviation. ^*^P<0.05, compared with the sham group. VEGF, vascular endothelial growth factor.

The content of VEGF in irradiated brain tissue was quantified by ELISA. At 4, 8, 12, 16, 20 and 24 weeks post radiation, the concentration of VEGF in the radiation group was 5.51±0.36, 7.70±1.16, 9.12±0.76, 12.95±2.13, 11.44±1.57 and 6.36±0.97 pg/ml, respectively, as compared with 5.36±0.60, 5.56±0.61, 5.55±0.84, 5.83±0.52, 6.05±0.59 and 5.50±0.87 pg/ml in the cortex of the right hemisphere of the sham rats. The expression of VEGF was significantly higher in rats with GKS compared with the controls at 8, 12, 16 and 20 weeks after GKS (P<0.01; Fig. 2). Over the subsequent 8 weeks, however, VEGF expression in the rat brain tissue receiving GKS began to decrease and recovered to levels that were similar to those recorded in the normal nonirradiated cortex. No difference was observed between the radiation groups and controls at 4 and 24 weeks after GKS (P=0.82 and 0.14; Fig. 2).

### qPCR analysis

**Figure 3:**
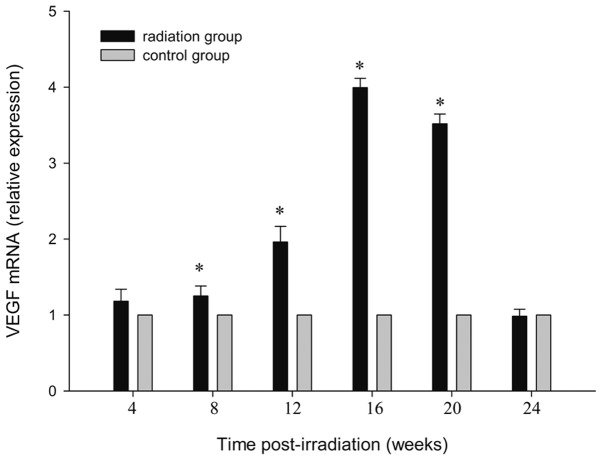
Time course of quantitative polymerase chain reaction analysis of VEGF mRNA following GKS in irradiated tissue. The data are presented as the mean ± standard deviation. ^*^P<0.05, compared with the sham group. VEGF, vascular endothelial growth factor.

Using qPCR analysis, mRNA from irradiated brain tissue was examined 4, 8, 16, 24, 20 and 24 weeks after irradiation and compared with the sham-irradiated controls. At 4 weeks after irradiation, a statistically significant upregulation was detected (P<0.01). The highest expression of VEGF mRNA was observed at 16 weeks post-irradiation (P<0.001), and returned to the control at 24 weeks post radiation (P=0.71). Data up to 24 weeks after irradiation are shown in Fig. 3. By contrast, a few differentially expressed genes in the cortex of the control rats were observed (P=0.98). The steep response in the VEGF mRNA essentially paralleled that for protein.

### Vessel density

**Figure 4:**
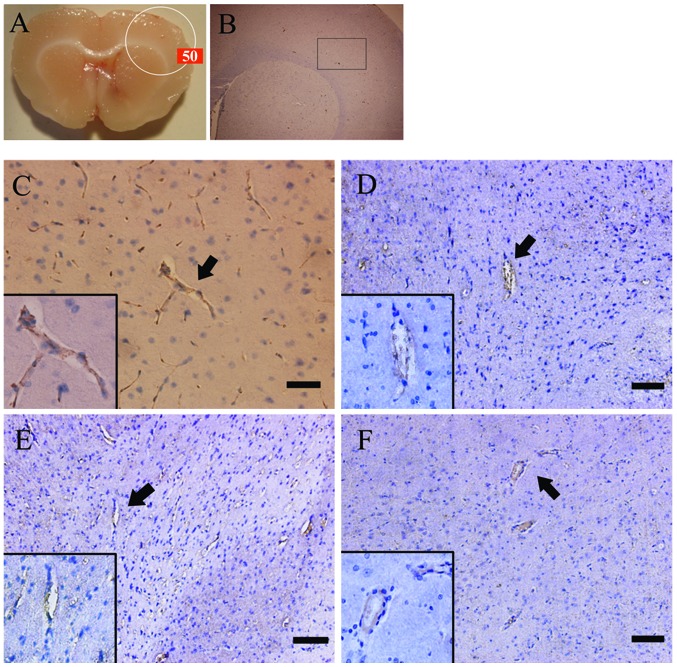
Angiogenesis in the irradiated cortical area as demonstrated by immunochemistry. (A) Coronal section of the rat brain showing the 50% isodose curve with a maximal center dose of 60 Gy. (B) Microvascular morphology and density were observed by light microscopy in the area of the center maximum dose. Magnification, ×40. (C) Presentative immunohistochemistry fields of CD31 (endothelial marker) in the cortex of the normal control. Magnification, ×200 (D) 4 weeks post irradiation, vessel density was significantly reduced in irradiated tissue. Magnification, ×100 (E) Vessel density was moderately increased 16 weeks later compared with the controls and vacuolization was observed in the irradiated tissue. Magnification, ×100 (F) 24 weeks post irradiation the vessel density was reduced to control levels in irradiated tissue. Magnification, ×100. Scale bars=150 μm in B, C and D.

**Figure 5:**
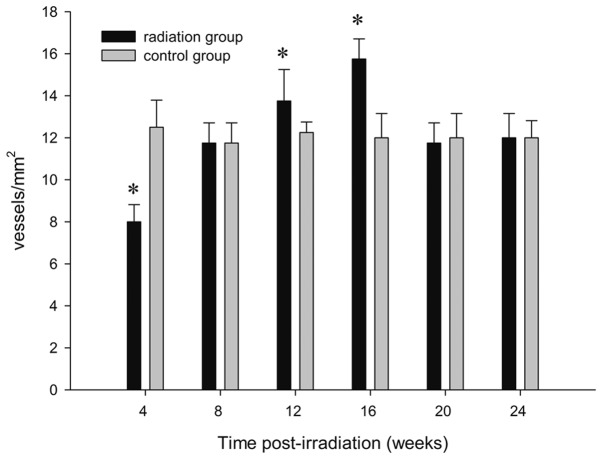
Bar graph representing the number of endothelial cells (positive for CD31) per highpower field. The average signal for each animal (based on five areas) is plotted over 6 months, showing a difference between GKS-treated and control animals. The data are presented as the mean ± standard deviation. ^*^P<0.05, compared with the sham group.

To determine whether the changes in angiogenesis occurred in the radiation target, CD31^+^ cell counts were assessed in the sham and radiation group. Sham animals had an average vessel density of 12.50±1.29 vessels/mm^2^ 4 weeks after sham injury (Fig. 4C). The results in the sham control rats were not significantly different at any time point following the procedure (P=0.38). As compared with the control level, a decrease in cell counts was observed 4 weeks after GKS (8.00±0.82 vessels/mm^2^, P=0.02; Figs. 4D and 5). At a later time, CD31^+^ cell counts increased slowly. It was significantly higher in the cortex of irradiated rats compared with that of the control rats 12 weeks post-irradiation (13.75±1.50 vessels/mm^2^; P<0.001; Fig. 5), reached acme 16 weeks after GKS (15.75±0.96 vessels/mm^2^; P<0.001; Figs. 4E and 5) and then gradually decreased to the control level (12.00±1.16 vessels/mm^2^; P=0.55; Figs. 4F and 5) 24 weeks after GKS.

### Electron microscopy

**Figure 6:**
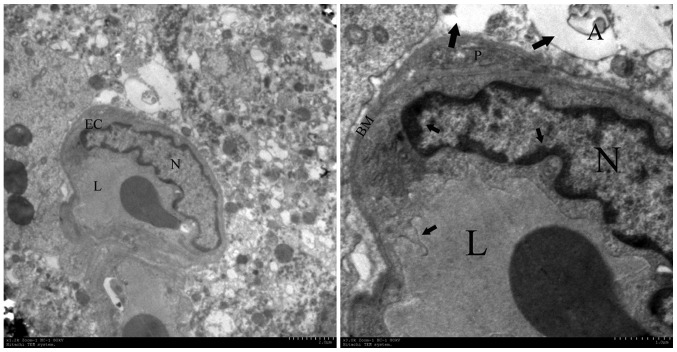
Ultrastructural observations of brain capillary endothelial cells. The most pronounced alterations were observed in disturbed vessels and perivascular structures in the irradiated tissue at week 16, which contained enlargement of endothelial nuclei, pykno-chromatin (short arrow), thickening of the capillary basement membrane, folding of the plasma membrane (short arrow) and astroglial edema (long arrow). A, astrocyte; EC, endothelial cells; N, nuclei, L, capillary lumen; BM, basement membrane. Magnification: Left, ×10,000; right, ×50,000.

Ultrastructural changes of Gamma-irradiated hemispheres of rat brains were detected by transmission electron microscopy. The abnormal brain endothelial cells and surrounding tissue were demonstrated in the irradiated cortex 16 weeks post radiation (Fig. 6). Chromatin condensation and aggregation were observed at the periphery of the nucleons. The membranous structures demonstrated destructive changes, including the thickened basement and the folding of the plasma membrane. The swelling of astrocytic perivascular processes were also observed around damaged endothelial cells. The perivascular cells showed the presence of vacuolar-disturbed structures in the matrix.

### Brain water content

**Figure 7:**
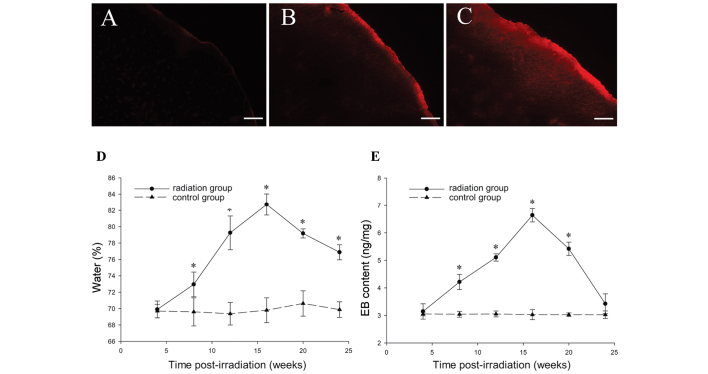
Representative results of brain water content and EB extravasation performed between 4 and 24 weeks after GKS. EB extravasation in the cortex area ipsilateral to radiation was identified by fluorescence microscopy (A) 4, (B) 8 and (C) 16 weeks after GKS. The dynamic changes in the (D) brain water content and (E) EB extravasation over time in irradiated tissues or controls are shown. Scale bar=100 μm. The data are presented as the mean ± standard deviation. ^*^P<0.05, compared with the sham group. EB, Evans Blue; GKS, Gamma knife surgery.

An increase in brain water content was observed in irradiated tissue (Fig. 7D) 8 weeks post-irradiation as compared with the sham controls (P=0.03). The highest brain water content was observed 20 weeks post-radiosurgery in the irradiated tissue (82.73±1.30%; P<0.001), following which the content gradually decreased; however, it remained higher than the control levels (P<0.001). No change in brain water content was detected in any of the sham-irradiated control animals at various intervals (P=0.85).

### EB extravasation experiment

No EB fluorescence was detected in the right parietal cortex 4 weeks after GKS (Fig. 7A). EB fluorescence was only observed in the irradiated cortex (Fig. 7B) 8 weeks following GKS, and appeared to be significantly stronger 16 weeks after GKS (Fig. 7C). The EB extravasation in the radiation and sham surgery groups was quantified as ng of EB per milligram of brain tissue at 4, 8, 12, 16, 20 and 24 weeks after GKS. The EB concentration in the radiation tissue was significantly higher than in the sham operation group at 8, 12, 16 and 20 weeks after GKS (P<0.001; Fig. 7E). Although the content demonstrated a maximum level 16 weeks after GKS (6.64±0.24 ng/mg; P<0.001; Fig. 7E), there was a significantly steep slope of EB content in the radiation group between 20 and 24 weeks after GKS. No differences were identified between the radiation and sham groups 24 weeks after GKS (P=0.09; Fig. 7E).

## Discussion

In the field of neurological surgery, GKS is becoming an indispensable treatment means of various brain disorders. The radiation dose that can be administered safely is nearly always limited by the risk of radiation injury to normal CNS tissue. An understanding of the mechanisms of normal tissue injury following GKS may have important implications and applications in the development of radiation modulators or treatments to reduce the consequences of a given radiation exposure.

The protein level of VEGF is overexpressed in a variety of CNS diseases, including tumor, ischemia and traumatic brain injury (17–19). In intraoperative radiotherapy, the expression of VEGF in the irradiated cerebral hemispheres was significantly increased within 8 weeks in the radiation group (20). Furthermore, in the rat spinal cord, the induction of the expression of VEGF by a high dose of 22 Gy X-ray has been previously demonstrated (21). However, alterations in VEGF in response to GKS remain largely unknown. The present study disclosed marked VEGF-positivity in the radiated normal brain following GKS in the subacute stage. In this series, VEGF expression induced by radiosurgery was time dependent. Its expression in irradiated regions gradually increased over a period of 8 weeks, reached maximal expression 16 weeks after radiosurgery and started to decrease 20 weeks after radiosurgery. The mRNA expression in irradiated tissue was also observed between 4 and 20 weeks after GKS, which was similar to that of the protein expression. The early changes of VEGF following conventional radiotherapy and radiosurgery are similar, but the increased VEGF following radiosurgery sustained longer time. Radiosurgery-induced delayed damage to the normal brain can occur several months following treatment. This later decrease in VEGF expression was likely associated with delayed cell loss or necrosis. VEGF is important in angiogenesis, which is either a protective factor or a response to injury. VEGF drives the formation of new blood vessels in various neurological disorders, including tumors, stroke and traumatic brain injury (22–24). In the current study, this dynamic change in VEGF was likely attributable to pathological angiogenesis following GKS.

Several experiments have demonstrated endothelial cell dynamics in the rat brain following local irradiation. For example, it has been demonstrated that a decrease in endothelial cell number was observed within 1 day of irradiation with doses of 25 Gy, an abortive recovery occurred at ~25 weeks, and then decreased to 50% by the time white matter necrosis became evident (25). By contrast, a tendency towards an increase in the average cross-sectional area of the vessels in irradiated regions occurred 1 month after 75 Gy radiation, and 1 week after 120 Gy radiation (14). In the current study, the loss of endothelial cells appeared 4 weeks after GKS compared with the controls. Subsequently, a slight vessel density increase was observed 12 weeks after radiation; this effect became clear 16 weeks after GKS and then gradually decreased to the control level. The change in angiogenesis was consistent with the change in VEGF expression. This suggested an important role for VEGF in the angiogenesis of GKS injury; however, the results cannot suggest that VEGF has a protective effect on GKS injury in rats, as VEGF may also have detrimental effects, including an increase in endothelial permeability, which is recognized as a cardinal feature of pathological angiogenesis. A previous study demonstrated that there were pathological vascular alterations in the irradiated field following GKS, which included increased vascularity, edema and fibrin exudation (26). In the present study, the swollen endothelial cell, thickened basement membrane and severely edematous end-feet of glial cells in irradiated areas could be observed 16 weeks after radiation. This morphological alteration indicated that there could be pathological angiogenesis in the radiation-damaged region. In this condition, the blood brain barrier function was likely altered, resulting in a subsequent increase in vascular permeability.

VEGF-induced increases in microvessel permeability have been demonstrated not only in normal brain endothelial cells, but also in diseased conditions, including in tissue surrounding brain tumors (27) and the ischemia lesion area (28). In addition, the upregulation of VEGF in the irradiated spine was present in regions where increased permeability was observed (16). In the present study, the EB extraction ratio also increased with time, attaining a plateau value in ~16 weeks and then declining toward control levels. Increases in brain water content were initially observed 8 weeks after irradiation, increased sharply to maximal value 16 weeks after radiosurgery treatment and then declined at 20 weeks. The maximal expression of VEGF coincided with the peak value of brain edema and EB extraction ratio. This is also similar to a previous study where VEGF expression in the spinal cord was present 16–20 weeks after 22 Gy irradiation, when significant blood spinal cord barrier breakdown is observed (29). By contrast, EB extravasation was observed in the irradiated cortex with higher VEGF expression, and no VEGF expression or changes in EB extravasation were detected in nonirradiated regions in animals receiving radiosurgery and the cortex in the controls. The temporal and spatial association of increased VEGF protein and vascular lesions suggests that VEGF upregulation is associated with increases in vascular permeability and edema formation resulting from Gamma knife radiation injury.

In contrast to VEGF and EB extraction, brain water was at a higher level between 20 and 24 weeks after GKS, although it began to decrease. Considering the complexity of edema formation and pathological effects of radiation, the current experiments cannot fully clarify this result. Previous studies have demonstrated that histological changes following radiation are time dependent (14). The vascular response occurred shortly following radiation, while necrosis and loss of cells appeared following a more prolonged post-radiosurgical interval (30). The delayed tissue injury is likely to be initiated by capillary modifications leading to microcirculatory disturbance. Although VEGF was at a lower level 24 weeks following GKS, early VEGF increases and vascular abnormality were likely associated with late edema formation.

In conclusion, our data revealed that the expression of VEGF in the irradiated cortex was significantly dynamic over time following the subacute period, when abnormal angiogenesis and alterations in vascular permeability were observed. These results indicated that the alterations in VEGF expression in the irradiated tissue may be an important cause of the development of pathological angiogenesis and cerebral edema following GKS. The time course of expression of VEGF may provide important dependencies for therapeutic opportunity of GKS radiation injury. The aim of our future experiments is to identify crucial molecular mechanisms in this event that may potentially cause gamma knife radiation injury.
